# Development of a programmed cell death ligand-1 immunohistochemical assay validated for analysis of non-small cell lung cancer and head and neck squamous cell carcinoma

**DOI:** 10.1186/s13000-016-0545-8

**Published:** 2016-10-08

**Authors:** Marlon C. Rebelatto, Anita Midha, Amita Mistry, Constantine Sabalos, Nicole Schechter, Xia Li, Xiaoping Jin, Keith E. Steele, Paul B. Robbins, John A. Blake-Haskins, Jill Walker

**Affiliations:** 1 MedImmune, One MedImmune Way, Gaithersburg, MD 20878 USA; 2 AstraZeneca, Alderley Park, Macclesfield, UK; 3 Ventana Medical Systems Inc., Tucson, AZ USA; 4 AstraZeneca, Cambridge, UK

**Keywords:** Durvalumab, MEDI4736, PD-L1, Diagnostic, Immunohistochemistry, Assay, NSCLC, HNSCC, Immunotherapy

## Abstract

**Background:**

A high-quality programmed cell-death ligand 1 (PD-L1) diagnostic assay may help predict which patients are more likely to respond to anti-programmed cell death-1 (PD-1)/PD-L1 antibody-based cancer therapy. Here we describe a PD-L1 immunohistochemical (IHC) staining protocol developed by Ventana Medical Systems Inc. and key analytical parameters of its use in formalin-fixed, paraffin-embedded (FFPE) samples of non-small cell lung cancer (NSCLC) and head and neck squamous cell carcinoma (HNSCC).

**Methods:**

An anti-human PD-L1 rabbit monoclonal antibody (SP263) was optimized for use with the VENTANA OptiView DAB IHC Detection Kit on the automated VENTANA BenchMark ULTRA platform. The VENTANA PD-L1 (SP263) Assay was validated for use with FFPE NSCLC and HNSCC tissue samples in a series of studies addressing sensitivity, specificity, robustness, and precision. Samples from a subset of 181 patients from a Phase 1/2 study of durvalumab (NCT01693562) were analyzed to determine the optimal PD-L1 staining cut-off for enriching the probability of responses to treatment. The scoring algorithm was defined using statistical analysis of clinical response data from this clinical trial and PD-L1 staining parameters in HNSCC and NSCLC tissue. Inter-reader agreement was established by three pathologists who evaluated 81 NSCLC and 100 HNSCC samples across the range of PD-L1 expression levels.

**Results:**

The VENTANA PD-L1 (SP263) Assay met all pre-defined acceptance criteria. For both cancer types, a cut-off of 25 % of tumor cells with PD-L1 membrane staining of any intensity best discriminated responders from nonresponders. Samples with staining above this value were deemed to have high PD-L1 expression, and those with staining below it were deemed to have low or no PD-L1 expression. Inter-reader agreement on PD-L1 status was 97 and 92 % for NSCLC and HNSCC, respectively.

**Conclusions:**

These results highlight the robustness and reproducibility of the VENTANA PD-L1 (SP263) Assay and support its suitability for use in the evaluation of NSCLC and HNSCC FFPE tumor samples using the devised ≥25 % tumor cell staining cut-off in a clinical setting. The clinical utility of the PD-L1 diagnostic assay as a predictive biomarker will be further validated in ongoing durvalumab studies.

**Trial registration:**

ClinicalTrials.gov: NCT01693562

This article provides the full results of two abstracts that have previously been presented and published [1, 2].

## Background

Patients with recurrent and metastatic non-small cell lung cancer (NSCLC) and head and neck squamous cell carcinoma (HNSCC) have considerable unmet medical needs and require improved treatments with the potential to provide prolonged survival and reduced exposure to toxic chemotherapies [3–5].

It has long been recognized that the immune system is capable of recognizing cancer as foreign and developing a specific immune response. A number of stimulatory and opposing inhibitory proteins have been shown to regulate the quality and magnitude of the antitumor response [6–9]. For example, tumors can evade detection by the immune system by exploiting one or more of the inhibitory (checkpoint) pathways that suppress antitumor T-cell responses [9]. The therapeutic blockade of these checkpoint molecules is actively being explored across different cancers [6, 7]. One of the most promising approaches is antibody blockade of the programmed cell death-1 (PD-1)/ programmed cell death ligand-1 (PD-L1) pathway [6, 7]. The anti-PD-1 agents nivolumab and pembrolizumab have demonstrated clinical activity across several tumor types and are now approved for various indications worldwide [10–13]. While dramatic responses have been seen in a number of patients treated with anti-PD-1 and anti-PD-L1 antibodies, responses have typically represented only a fraction of treated patients. This has led to a further search for biomarkers predictive of response. Chief among these is the demonstration of PD-L1 in tumor tissues using immunohistochemistry (IHC). Two different IHC assays have so far been approved by the Food and Drug Administration (FDA) for use as diagnostic tests in advanced NSCLC; a complementary diagnostic for nivolumab [14] and a companion diagnostic for pembrolizumab [15].

PD-L1 (B7-H1, CD274) is part of a complex system of receptors and ligands that are involved in regulating T-cell activation. Its main function is to regulate the balance between T-cell activation and tolerance through interaction with its two receptors, PD-1 (CD279) and CD80 (B7-1). In normal tissue, PD-L1 has been reported to be expressed on a subset of T cells, B cells, dendritic cells, macrophages, mesenchymal stem cells, and bone marrow-derived mast cells, as well as various non-hematopoietic cells [16, 17]. PD-L1 is expressed in a limited set of normal epithelial cells, such as placental trophoblast cells and crypt epithelium of the tonsil [18]. Its expression in these locations is thought to provide protection from cell-mediated attack. Importantly, and in a similar fashion, PD-L1 is also expressed in a broad range of carcinomas and other cancers [19–22] including NSCLC and HNSCC [9, 16]. In the tumor microenvironment, PD-L1 expressed on tumor cells binds to PD-1 on activated T cells reaching the tumor. This delivers an inhibitory signal to those T cells, preventing them from killing target tumor cells, and protecting the tumor from immune elimination [9].

PD-L1 biology is further complicated in additional ways. For example, full-length PD-L1 is composed of an extracellular domain that contains the PD-1 binding domain, a shorter intracellular domain, and a short transmembrane region. A number of antibodies to the extracellular and intracellular domains have been generated and several of those appear useful as IHC reagents [22–24]. It is also the case that splice variants of PD-L1 have been identified [25, 26] and individual isoforms may localize to the cytoplasm or membrane of cells in vitro. It should be noted, however, that particular forms of the PD-L1 protein may be recognized by some but not all of the other available antibodies. Cytoplasmic or membrane immunolabeling of neoplastic cells and macrophages have also been shown in tumor tissues [19, 27]. PD-L1 localization to the cell membrane is likely required for interaction with PD-1. In addition, PD-L1 demonstrates a range of apparent expression levels in cells, whether cytoplasmic or membranous. Tumor cells or immune cells with variable intensities of PD-L1 expression could therefore suppress PD-1-expressing T lymphocytes to different degrees, although this has not yet been proven. In addition to the biological heterogeneity of PD-L1 expression (notably intra- and inter-tumoral and temporal variations), [28, 29] there is also technical variability due to a number of different assays (developed for diagnostic or research use) being used to label PD-L1 in tumor tissue. These have variable target specificity and selectivity (being raised to different epitopes on the PD-L1 molecule), and use different methods for antibody detection and approaches to determining PD-L1 expression (scoring systems and staining cut-offs) [28].

Not surprisingly then, given the various complexities noted, substantial discordance is seen among published studies relying on PD-L1 IHC [22–24]. Nonetheless, the ability of a PD-L1 IHC method to account for those key aspects of PD-L1 biology and tissue expression relevant to therapy with either PD-1 or PD-L1 antibodies is expected to substantially contribute the overall usefulness of the assay. In that context, for example, an assay that demonstrates distinct cell membrane staining and a dynamic range of PD-L1 expression is highly desired. In addition, the ability to optimally label PD-L1 on neoplastic cells as well as immune cells is important, as is the ability to perform across the range of tissue specimens upon which the assay is expected to be applied. These qualities are especially needed to increase the ability to accurately identify patients who may respond to immunotherapies targeting PD-L1 or PD-1. At the same time, they contribute to a more comprehensive and potentially accurate assessment of PD-L1 that reflects the complexities of its expression in a range of tumor types and that may be required to clarify aspects of its immunosuppressive role in cancer that are not yet fully understood.

Durvalumab (MEDI4736) is a selective, high-affinity human IgG1 monoclonal antibody that blocks PD-L1 binding to PD-1 and CD80 but does not bind to programmed cell death ligand-2 (PD-L2) [30]. PD-L2 plays a role in controlling inflammation in normal lung tissue (with expression in lung macrophages and antigen-presenting cells) [31] and this may help to avoid PD-L2-mediated immune-related toxicities, which have been observed in animal models [32, 33]. In an ongoing Phase 1/2 study of patients with advanced solid tumors, durvalumab monotherapy has demonstrated a manageable tolerability profile and encouraging antitumor activity across multiple tumor types, including NSCLC and HNSCC [34, 35].

Most NSCLC tumors do not express PD-L1 at high levels, although reported levels vary; [20, 36, 37] only ~20 % of NSCLC tumors were reported to show PD-L1 expression in 5 % or more of tumor cells obtained with three different IHC assays (using different PD-L1 antibody clones; E1L3N, 22C3, and 5H1) [20, 38, 39]. In HNSCC, tissue PD-L1 immunostaining increases vs. normal controls [27] with variable levels of PD-L1 expression [18, 35, 40]. The absence of detectable PD-L1 in some tumors may account for poor responses to anti-PD-1/PD-L1 therapy. A sensitivity analysis of the clinical activity of nivolumab, pembrolizumab, and the anti-PD-L1 agent atezolizumab in NSCLC [41–48] demonstrated that overall response rates were significantly lower in patients with PD-L1 low tumors (1–5 % tumor cells stained using various PD-L1 assays) than in patients with PD-L1 high tumors [42]. Evidence from some individual clinical studies of the anti-PD-1/PD-L1 agents nivolumab, durvalumab, pembrolizumab, atezolizumab, and avelumab also suggest that patients with PD-L1 high tumors can experience improved treatment benefits vs. those with PD-L1 low tumors [34, 43, 47, 49, 50].

The development and application of diagnostic tests in clinical practice to identify patients most likely to benefit from anti-PD-1/PD-L1 therapy could improve patient outcomes and decrease healthcare costs, while directing patients that are less likely to respond towards other alternative treatment options. Research into the relative performance of different PD-L1 assays and their reliability for detecting PD-L1 is ongoing [51]. These diagnostic tests, which are designed to aid treatment decisions with specific anti-PD-1 therapies [23, 29, 52], are likely to be among the key drivers for the future of personalized health care in oncology [23].

In this paper, we describe the development and validation of the VENTANA PD-L1 (SP263) Assay (PD-L1 [SP263]) (Ventana Medical Systems Inc., Tucson, AZ, USA), which has been designed for the detection of PD-L1 protein in formalin-fixed, paraffin-embedded (FFPE) NSCLC and HNSCC tumor samples. In addition, we describe the identification and validation of scoring criteria (defined with tissues from a durvalumab clinical trial) that can be used to classify samples as PD-L1 high expression or PD-L1 low/no expression in NSCLC and HNSCC tissue.

## Methods

### PD-L1 (SP263) assay

#### Antibody generation

The PD-L1 (SP263) assay uses an anti-human PD-L1 rabbit monoclonal antibody (SP263) directed against the cytoplasmic region of human PD-L1. This peptide was synthesized and conjugated to the carrier protein keyhole limpet hemocyanin. New Zealand White rabbits were immunized with keyhole limpet hemocyanin conjugated peptide emulsified with complete Freund’s adjuvant followed by a series of booster doses of immunogen emulsified with incomplete Freund’s adjuvant. The rabbit that generated IHC-positive polyclonal antibody was then selected for monoclonal antibody development using Spring Bioscience’s proprietary technology.

Briefly, antibody-expressing cells were isolated and screened by enzyme-linked immunoabsorbent assay for reactivity to the immunogen and by IHC on control tissue blocks. Complementary DNA coding for antibody heavy chains and light chains was isolated from IHC-positive clones. Monoclonal antibodies were produced by co-transfection of heavy- and light-chain complementary DNA and tested again by IHC. The clone producing antibodies with the best specificity was selected. A larger transfected production lot was produced and the monoclonal antibody was purified through a Protein A column.

#### Staining protocol

The antibody was designed to be used in conjunction with the VENTANA OptiView diaminobenzidine tetrahydrochloride (DAB) IHC Detection Kit (P/N 760-700) and the staining protocol for the assay is provided in Table 1. This protocol represents the selectable options within the detection kit procedure. The OptiView DAB IHC Detection Kit is an indirect, biotin-free system for detecting mouse IgG, mouse IgM, and rabbit primary antibodies used to detect target antigens in FFPE tissue, The 3-hydroxy-2-quinoxalinecarbamide (hydroxyquinoxaline) universal linker conjugated with hydroxyquinoxaline haptens binds to the primary antibody and is recognized by the horse radish peroxidase multimer. The remaining components in the detection kit function to create a brown 3,3’-DAB precipitate on the tissue section at the site of PD-L1 antigen.

**Table 1:** Recommended staining protocol for VENTANA PD-L1 (SP263) Assay (OptiView DAB IHC kit, BenchMark ULTRA instrument)

Procedure type	Method
Deparaffinization	Selected
Cell conditioning (Antigen unmasking)	Cell conditioning 1, 64 min
Pre-primary peroxidase inhibitor	Selected
Antibody (primary)	16 min, 36 °C
OptiView HQ linker	8 min (default)
OptiView HRP multimer	8 min (default)
Counterstain	Hematoxylin II, 4 min
Post counterstain	Bluing, 4 min

*DAB* 3, 3’-diaminobenzidine tetrahydrochloride*, HRP* horse radish peroxidase, *HQ* hydroxyquinoxaline, *IHC* immunohistochemistry, *PD-L1* programmed cell death ligand-1

Normal-term placenta can be used as a positive and negative tissue control for the assay. Tissue controls were used to monitor the correct performance of processed tissues, test reagents, and instruments. One placenta control was included on each staining run.

#### Cell line analysis of PD-L1 expression

The SP263 antibody was tested by immunocytochemistry on the following cell lines: Calu-3, KARPASS 299, H820, H1975, MDA-MB231, T-47D, LOX, ACHN, MCF-7, and HCT-116. In addition, HEK293 cell lines transfected to express varying levels of PD-L1 were prepared to test PD-L1 expression across the dynamic range and were also transfected to express PD-L2 to demonstrate antibody specificity.

#### Flow cytometry analysis

Tumor cell lines (LOX, MCF-7, MDA-MB231, HCT116, and ACHN) were evaluated for surface PD-L1 expression and the number of receptors per cell was estimated using flow cytometry. Briefly, tumor cell suspensions were incubated with 100 μl of anti-human PD-L1 antibody (R&D systems, catalog MAB1561) diluted in flow cytometry analysis (FACS) buffer (phosphate-buffered saline plus 2 % heat inactivated fetal bovine serum) for 45 min at 4 °C. After primary monoclonal antibody incubation, cells were washed with cold FACS buffer and resuspended in 100 μl QIFI Kit FITC secondary antibody diluted 1:50 with FACS buffer (Dako QIFI Kit, catalog #K0078, lot 00088291). Secondary detection antibody incubation was conducted for 45 min at 4 °C, protected from light. After secondary incubation, cells were washed once with cold FACS buffer and resuspended in FACS buffer for flow cytometric analysis performed on a BD LSR II Flow Cytometer (BD Biosciences, Mountain View, CA, USA). Using the setup provided in the QIFI kit, a standard curve was plotted using the mean fluorescent intensity values and calculated using GraphPad Prism 6 software. The x values were determined, which correlated to the number of receptors per cell.

#### Western blot analyses of cell lysates

Western blot studies were conducted by SDS-PAGE. Cell lysates were prepared from four different cell lines that demonstrated varying IHC protein expression (H820, MDA-MB231, H1975, and Calu-3 cell lines). A recombinant human PD-L1 protein served as a positive control and a recombinant BCL-2 protein served as a negative control for the study. An anti-actin antibody (8H10D10) (Cell Signaling Technologies, Danvers, MA, USA) was used to detect a ~42 kD protein actin. This constitutively expressed reference protein ensured equivalent loading of samples onto the gel.

#### Staining of commercially available normal and tumor tissue samples

Normal and tumor tissue array samples (Tissue Microarray FDA808ci, US Biomax, Rockville, MD, USA) were stained with the PD-L1 (SP263) rabbit monoclonal antibody using the final optimized protocol on the BenchMark ULTRA. A rabbit monoclonal negative-control Ig was also analyzed for the array staining run.

#### Evaluation of PD-L1 staining on tumor samples

All tumor sample evaluations were conducted by board-certified pathologists at Ventana’s College of American Pathologists accredited and Clinical Laboratory Improvement Act certified laboratory. Upon receipt of each sample, hematoxylin and eosin staining was performed to determine the number of tumor cells. The sample was considered acceptable for further analysis if it contained ≥100 viable tumor cells. The PD-L1 (SP263) assay was carried out on 4–6 μm sample sections mounted onto positively charged slides. PD-L1 staining was evaluated by estimating the percentage of cells stained at different intensities, from 0 to 3, with 0 representing no staining and 3 representing strong staining. This percentage was determined for staining localized in the cytoplasm and membrane of tumor cells. In addition, PD-L1 staining in tumor-associated immune cells (macrophages, dendritic cells, lymphocytes, etc.) was evaluated as a percentage of immune cells present in the tumor area.

#### PD-L1 scoring algorithm: Statistical analysis to identify the cut-off for determining PD-L1 positive/negative tumors

In NSCLC and HNSCC samples, PD-L1 was generally expressed as a continuum, ranging from a few tumor cells and/or immune cells expressing PD-L1 to the majority of tumor cells and immune cells expressing the target. Intensity of staining also varied, with mixtures of weak, moderate and intensely-stained tumor, and immune cells sometimes seen in single tumor specimens. To understand the association of the biomarker with clinical response, patient samples need to be classified into categories. For these purposes, samples were classified into two categories: high and low/no expression. The optimal scoring criteria were determined by Maximal Chi-square [53] and receiver operating characteristic (ROC) curves [54] of the PD-L1 staining parameters relative to clinical response in a subset of 155 patients with NSCLC enrolled in the NCT01693562 clinical trial [1, 34, 35]. The cut-off determination for the PD-L1 (SP263) assay was made following retrospective evaluation of their tumor samples as well as commercially available tumor samples. The considerations for the selected cut-off included prevalence, clinical outcome and the reproducibility of the test.

The endpoints used for the actual cut-off determination were the confirmed objective response (Y) per RECIST v1.1 based on investigator assessment and the percentage tumor cell membrane positivity for PD-L1 (X).

This scoring algorithm and the selected cut-off were utilized in the definitions of PD-L1 high and PD-L1 low/negative in the subsequent verification studies.

### PD-L1 (SP263) assay verification studies

#### Cut-slide stability

Cut-slide stability was evaluated on four tissue samples stained with the recommended assay protocol after storage under two different conditions (2–8 and 30 °C). Cut-slide stability was also assessed on slides stained at various timepoints after sectioning. Slides sectioned and stained at the Day 0 timepoint served as the comparator for the remainder of the tested timepoints (Day 3, 14, and monthly from Month 1–12 for NSCLC and Day 3, 14, and monthly from Month 1–10 for HNSCC).

#### Tissue thickness

Tissues were sectioned at various thicknesses (3–7 μm) to assess the effect of thickness on the staining performance of the PD-L1 (SP263) assay. The placenta tissue positive-control slides included in the staining run were sectioned at 4 μm. Slides were required to stain within 1.0 intensity point of each other and to maintain the same PD-L1 status (high or low/no expression).

#### Fixation type and time and ischemia

The effects of fixative type and fixation time were evaluated in tonsil-positive control tissue. Tonsil cases were processed in six fixatives (10 % neutral-buffered formalin [NBF], zinc formalin, 95 % alcohol, acidified formal alcohol (AFA), Z5 and Prefer) with multiple timepoints (1, 6, 12, 24, 72 h) in each fixative. The effects of ischemia were also investigated using KARPASS 299 xenograph tissues with ischemic times varying between 0, 0.5, 1, 2, 6, and 24 h. The immunostained slides were read and scored by a board-certified pathologist on a 0–3 intensity scale for specific PD-L1 staining and background. Data were then compared to the reference standard (10 % NBF for 24 h).

#### Intermediate precision

This design verification study was conducted to assess the PD-L1 (SP263) assay’s staining precision in NSCLC and HNSCC. Samples included both PD-L1 high and PD-L1 low/negative tissues across i) three different PD-L1 (SP263) antibody lots; ii) three OptiView DAB IHC Detection Kit lots; and iii) three BenchMark ULTRA instruments. This study was designed to verify the intermediate staining precision (inter-antibody lot, inter-detection kit lot, and intra-platform) of the PD-L1 (SP263) assay. A total of nine PD-L1 high and nine PD-L1 low/negative samples for each tumor type, spanning the range of PD-L1 expression, were used in the study.

#### Intra-day (slide to slide) precision

A total of 10 NSCLC and 10 HNSCC whole tissue samples (comprising five PD-L1 high and five PD-L1 low/negative samples for each tumor type) spanning the range of PD-L1 staining were evaluated across a cohort, which represented the dynamic range of PD-L1 expression. For intra-day repeatability, five replicate slides from each of the NSCLC or HNSCC specimens were stained on a single BenchMark ULTRA instrument (Tucson, AZ, USA) across two staining runs for NSCLC and HNSCC, respectively.

#### Intra-platform precision

A total of 10 NSCLC and 10 HNSCC samples (comprising five PD-L1 high and five PD-L1 low/negative samples for each tumor type) spanning the range of PD-L1 expression were evaluated across three BenchMark ULTRA instruments. Three staining runs were conducted for NSCLC and HNSCC, respectively.

#### Inter-day (day-to-day) precision

A total of 10 NSCLC and 10 HNSCC whole tissue samples (comprising five PD-L1 high and five PD-L1 low/negative cases for each tumor type) spanning the range of PD-L1 expression were evaluated. Two replicate slides were stained for each sample on three BenchMark ULTRA instruments on five non-consecutive days over a minimum span of 20 days.

#### Inter-laboratory precision

A total of 14 NSCLC and 14 HNSCC whole tissue samples (comprising seven PD-L1 high and seven PD-L1 low/negative cases for each tumor type) were evaluated. Cases were selected to cover the range of expression and included borderline cases around the cut-off. Each case was stained five different times in each laboratory. Three different laboratories were assessed. Two pathologists per laboratory conducted the scoring.

#### Reader verification of the scoring algorithm – inter- and intra-reader agreement

Between-reader (inter-reader) agreement with the PD-L1 (SP263) assay scoring algorithm was determined using a cohort of NSCLC tissue samples (previously screened for PD-L1 status) obtained from commercial sources and stained using the PD-L1 (SP263) assay. A total of 81 NSCLC samples (40 PD-L1 high and 41 PD-L1 low/negative) together with 100 HNSCC samples (50 PD-L1 high and 50 PD-L1 low/negative) were evaluated across the dynamic range of staining. Three readers were trained on the scoring algorithm and provided with the blinded and randomized study samples for scoring. Readers completed the initial scoring within 2 weeks and the scores were analyzed. There was a mandatory 2-week break period prior to the intra-reader precision scoring. Slides were then re-randomized and provided to the readers for intra-reader scoring.

## Results

### Antibody sensitivity and specificity

**Fig. 1:**
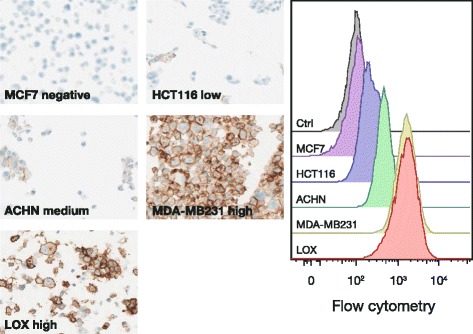
PD-L1 expression in adenocarcinoma cell lines labeled with the PD-L1 (SP263) antibody. Cell staining and flow cytometry results. Photomicrographs of cell lines stained with the PD-L1 (SP263) antibody (20X): MCF7 (negative control, no PD-L1 staining), HCT116 (low level of PD-L1 staining), ACHN (medium PD-L1 staining), MDA-MB231 (high-level PD-L1 staining), LOX (high-level PD-L1 staining). Corresponding fluorescence intensities achieved by flow cytometry with the same cell lines labeled with the PD-L1 (SP263) antibody. *Ctrl* control, *PD-L1* programmed cell death ligand-1

**Fig. 2:**
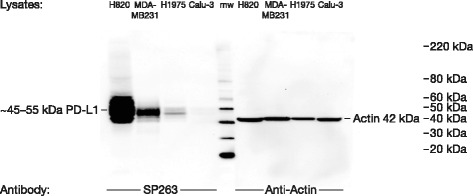
Western blot analyses of cell lysates labeled with the PD-L1 (SP263) antibody. Western blot analysis of cell lysates from adenocarcinoma cell lines loaded onto an SDS-PAGE gel and labeled with the PD-L1 (SP263) antibody. The antibody labeled a single band of the appropriate molecular weight for fully glycosylated PD-L1 (~55 kDa). Relative levels of PD-L1 immunoreactivity matched the known relative PD-L1 mRNA expression levels for these cell lines (H820 > MDA-MB231 > H1975 > Calu-3). *PD-L1* programmed cell death ligand-1

The SP263 antibody showed a high degree of sensitivity and specificity for PD-L1 in cell lines with various levels of PD-L1 expression, as determined by flow cytometry, immunocytochemistry (Fig. 1), and Western blot analysis (Fig. 2). The IHC protocol demonstrated a wide dynamic range, as indicated by intensities of staining of cell lines appropriate to their varying levels of PD-L1 expression. As Fig. 1 illustrates, immunolabeling ranged from minimal or mild in HCT116 (very low expression, ~1,600 molecules per cell) and ACHN cells, to moderate in MDA-MB231 cells, to intense in LOX (high expression, ~15,000 receptors per cell).

#### Western blot analysis of cell lysates

A single band of the appropriate molecular weight of fully glycosylated PD-L1 (~55 kDa) was detected by Western blot in the H820, MDA-MB231, and H1975 cell lines using the PD-L1 (SP263) antibody (Fig. 2). No band was detected for the Calu-3 negative-control cells. These results demonstrated the high specificity of SP263 for PD-L1.

Additional cell lines were examined using immunocytochemistry to further evaluate the specificity and sensitivity of the PD-L1 (SP263) assay for the PD-L1 analyte. In cell lines with differential native PD-L1-specific expression, PD-L1 staining intensity with the IHC assay corresponded to the relative levels of PD-L1 expression (LOX > ~MDA-MB231 > ACHN > HCT116 > MCF-7) (Fig. 1 and Table 2). In addition, human embryonic kidney cells (HEK293) were transfected to express varying levels of PD-L1 and examined for PD-L1 reactivity. HEK cells containing empty vector showed no immunolabeling, whereas the transfected HEK cell lines displayed differential PD-L1-specific labeling appropriate to their protein expression levels. The HEK293 cells transfected to express the closely-related PD-L2 protein had no detectable immunostaining by the PD-L1 (SP263) assay (Table 2).

**Table 2:** Cell lines with differential PD-L1 expression stained with the PD-L1 (SP263) antibody

	Membrane		Cytoplasm	
Cell line	% PD-L1^+^	Average intensity	% PD-L1^+^	Average intensity
MCF-7	0	NA	0	NA
HCT116	5	1.25	5	0.75
ACHN	50	1	0	NA
LOX	95	2.5	85	1.5
MDA-MB231	100	2.25	90	0.75
HEK293-vector	0	NA	0	NA
HEK293^−^ PD-L1^+^	5	3	5	3
HEK293^−^ PD-L1^++^	15	2.75	15	2.75
HEK293^−^ PD-L1^+++^	20	2.75	20	2.75
HEK293^−^ PD-L2^+++^	0	0	0	0

*NA* not applicable*, PD-L1* programmed cell death ligand-1, *PD-L2* programmed cell death ligand-2

#### Staining of commercially available normal and tumor tissue arrays

The PD-L1 immunostaining features of SP263 were further examined in a panel of normal human tissues, where staining was notable particularly in lymph node, tonsil, stomach (typically chief, or zymogenic cells), and placenta. As tonsil and lymph node are known to contain immune cells expressing PD-L1, staining in these tissues was expected. Trophoblast-lineage cell staining in placenta was also anticipated. No specific PD-L1 staining was observed in the parenchyma of a single normal lung core.

**Fig. 3:**
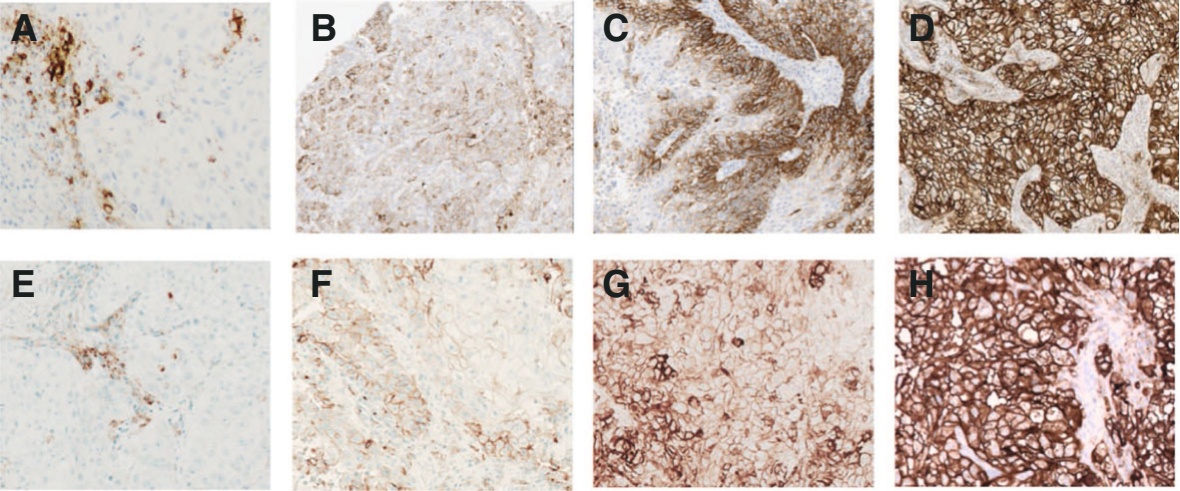
PD-L1 (SP263) antibody staining in tumor tissue samples. Photomicrographs of PD-L1 (SP263) staining in NSCLC and HNSCC tissue, demonstrating the range of PD-L1 staining intensity and frequency obtained with the PD-L1 (SP263) assay on NSCLC (**a**-**d**) and HNSCC (**e**-**h**) samples. **a**, **e** negative tumor cells (20X), positive immune cells; **b**, **f** (10X): low tumor staining; **c**, **g** (10X): moderate tumor staining; **d**, **h** (10X,20X): high tumor staining. *HNSCC* head and neck squamous cell carcinoma, *NSCLC* non-small cell lung cancer, *PD-L1* programmed cell death ligand-1

The following tumor tissue samples stained using the SP263 assay demonstrated PD-L1 immunostaining of tumor cells: squamous cell carcinoma of the skin, infiltrating ductal carcinoma of the breast, cholangiocarcinoma of the liver, Hodgkin lymphoma, squamous cell carcinoma of the lung, gastric adenocarcinoma, diffuse large B-cell lymphoma (in stomach), squamous cell carcinoma of the uterine cervix, endometrial adenocarcinoma, and thyroid papillary carcinoma. Most specimens also demonstrated immunostaining of tumor-infiltrating immune cells. The degree of apparent background staining was reported as ≤0.5 (faint) intensity on all evaluated cores, a level that generally did not interfere with specific PD-L1 labeling and therefore was judged to be acceptable. Additional commercial samples of NSCLC and HNSCC tissue showed similar PD-L1 labeling in a number of tumors. Figure 3 illustrates the range of SP263 staining intensities and frequencies in these tumors.

### PD-L1 scoring algorithm

**Fig. 4:**
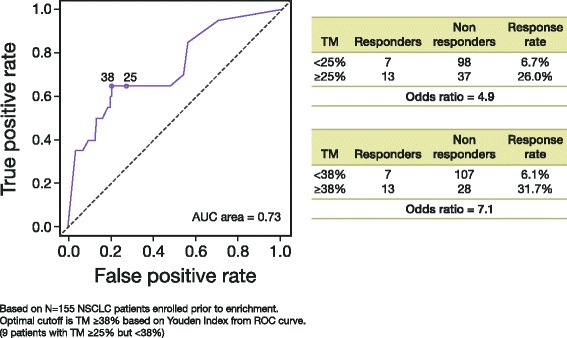
ROC analysis on confirmed objective response (per RECIST v1.1 per investigator). Analysis provided the rationale for the choice of tumor membrane score and 25 % cut-off. *AUC* area under the curve, *NSCLC* non-small cell lung cancer, *RECIST* Response Evaluation Criteria In Solid Tumors, *ROC* receiver operating characteristic, *TM* tumor membrane

**Fig. 5:**
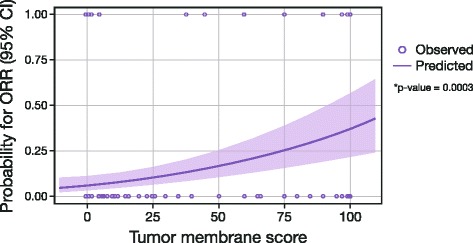
Relationship between ORR (per RECIST v1.1 per investigator) and tumor membrane score (logistic regression). *Based on *N* = 155 NSCLC patients enrolled prior to enrichment. *CI* confidence interval, *NSCLC* non-small cell lung cancer, *ORR* objective response rate, *RECIST* Response Evaluation Criteria In Solid Tumors

Clinical response to durvalumab and PD-L1 expression in tumor biopsies correlated best with PD-L1 expression in tumor cells rather than immune cells. In a subset of 155 patients with NSCLC in the Phase 1/2 trial, the optimal cut-off identified statistically (Fig. 4) was 38 %. However, based on other considerations (e.g., prevalence in the population, ease of scoring by pathologists, optimizing for higher negative predictive value), a cut-off of 25 % of tumor cells with membrane staining for PD-L1 at any intensity level above background was selected for the verification studies. Logistic regression showed a significant relationship between tumor membrane staining score and probability of response (*p*-value = 0.0003﻿, Fig. 5). Separate analysis of HNSCC samples from this study independently confirmed the reliability of the ≥25 % staining cut-off and exclusion of cytoplasmic tumor cell staining, immune cell staining, and staining intensity from the analysis of PD-L1 staining expression (data not shown).

### PD-L1 (SP263) assay verification study results

#### Cut-slide stability

The staining performance on NSCLC and HNSCC tissues stored at 2–8 and 30 °C passed the acceptance criteria. This test also demonstrated that staining at the Day 3 and 14, and Months 1–13 timepoints was consistent with results obtained on Day 0 for NSCLC tissues and staining at the Day 3 and 14, and Months 1–10 timepoints was consistent with results obtained on Day 0 for HNSCC tissues.

#### Tissue thickness and ischemia

Appropriate antibody staining was achieved across all tissue section thicknesses tested (3, 4, 5, 6, and 7 μm) (Table 3), which was consistent with PD-L1 high or low/negative outcomes for each case evaluated for both NSCLC and HNSCC. Variations in the types of tissue fixative used (either 10 % NBF, zinc formalin, or Z5) over time periods ranging from 6 to 72 h and cold tissue ischemia for periods ranging from 0 to 24 h also had a negligible effect on staining performance achieved with the PD-L1 (SP263) assay (Table 3). Fixation with 95 % alcohol, AFA and Prefer are not recommended for use with the assay as they caused a loss of staining intensity for PD-L1.

**Table 3:** Preanalytic factors

Study outline	Design	Results
Tissue thickness	NSCLC and HNSCC tissues sectioned at various thicknesses (3, 4, 5, 6, and 7 μm)	Antibody demonstrated appropriate staining across all thicknesses tested and was representative of clinical PD-L1 status for each case
Tissue fixation	Tonsil cases processed in 10 % NBF, zinc formalin, 95 % alcohol, AFA, Z5 and Prefer with multiple timepoints (1, 6, 12, 24, and 72 h) in each fixative	Timepoints (6, 12, 24, and 72 h) in 10 % NBF, zinc formalin and Z5 were acceptable; 95 % alcohol, AFA and Prefer were not acceptable
Ischemia	KARPASS 299 xenograft tissues with multiple cold ischemia times (0, 0.5, 1, 2, 6, and 24 h)	No significant change in staining intensity from time 0 to hour 24
Cut-slide stability	Four tissue samples; cut at 4 μm and stored at 2–8 and 30 °C. Stained at the Day 0 timepoint and at Day 3 and 14 and monthly Month 1 to 12 timepoints	Staining results at different storage temperatures and at timepoints Day 3 and 14, and Months 1 to 12 consistent with results achieved on Day 0

*AFA* acidified formal alcohol, *HNSCC *head and neck squamous cell carcinoma, *NBF* neutral buffered formalin, *NSCLC* non-small cell lung cancer, *PD-L1* programmed cell death ligand-1

#### Intra-day (slide to slide) precision, intra-platform precision, and inter-day (day-to-day) precision

The intra-day reproducibility of the PD-L1 (SP263) assay within a single staining run exceeded the 90 % pass criterion set for this study; overall percentage agreement (OPA) for PD-L1 high vs. low/no expression was 100.0 % with a 95 % confidence interval (CI) of 92.9–100.0. The positive percentage agreement was 100.0 % (95 % CI: 88.6–100.0) and the negative percentage agreement was 100.0 % (95 % CI: 83.9–100.0). The assay also exceeded the 90 % pass criteria set for intra-platform precision (performance across three BenchMark ULTRA instruments) and inter-day precision (performance on the same BenchMark ULTRA instrument over 5 non-consecutive days for a minimum of 20 days) (Table 4). All the tissues tested in these assay performance studies had acceptable background staining, which did not interfere with interpretation of PD-L1 high vs. low/negative results. Inter-lot variability of either antibody or DAB visualization reagents also had a negligible effect on assay performance (Table 4).

**Table 4:** PD-L1 (SP263) assay performance test results

Study outline	Design	NSCLC results, % (95 % CI)
Intra-day: performance within a single run on a BenchMark ULTRA instrument	10 cases 5 PD-L1 high 5 PD-L1 low/negative *n* = 100	PPA 100.0 (88.6–100.0) NPA 100.0 (83.9–100.0) OPA 100.0 (92.9–100.0)
Inter-day: performance on the same BenchMark ULTRA instrument over 5 non-consecutive days for a minimum of 20 days	10 cases 5 PD-L1 high 5 PD-L1 low/negative *n* = 50	PPA 100.0 (92.9–100.0) NPA 96.0 (86.5–98.9) OPA 98.0 (93.0–99.4)
Intermediate precision: Inter-antibody lot (*n* = 3 lots)	18 cases 9 PD-L1 high 9 PD-L1 low/negative PPA & NPA *n* = 243 OPA *n* = 486	PPA 99.2 (97.0–99.8) NPA 97.5 (94.7–98.9) OPA 98.4 (96.8–99.2)
Intermediate precision: Inter-detection kit lot (*n* = 3 lots)	18 cases 9 PD-L1 high 9 PD-L1 low/negative PPA & NPA *n* = 243 OPA *n* = 486	PPA 99.2 (97.0–99.8) NPA 97.9 (95.3–99.1) OPA 98.6 (97.1–99.3)
Intermediate precision: Intra-platform (*n* = 3 instruments)	18 cases 9 PD-L1 high 9 PD-L1 low/negative PPA & NPA *n* = 243 OPA *n* = 486	PPA 99.2 (97.0–99.8) NPA 97.5 (94.7–98.9) OPA 98.4 (96.8–99.2)

*CI* confidence interval, *NPA* negative percentage agreement, *NSCLC* non-small cell lung cancer, *OPA* overall percentage agreement, *PD-L1* programmed cell death ligand-1, *PPA* positive percentage agreement

#### Intermediate precision (inter-antibody lot, inter-detection kit lot, and intra-platform)

The intermediate precision design verification study was conducted to assess PD-L1 (SP263) assay staining precision on NSCLC tissues representing the clinical status range. The antibody exceeded the 90 % pass criteria set across the three antibody lots, three detection kit lots and three BenchMark ULTRA platforms (Table 4). All the tissues tested in these assay performance studies had acceptable background staining, which did not interfere with interpretation of PD-L1 high vs. low/negative results.

#### Intra-reader and inter-reader precision (agreement)

For NSCLC, the OPA between the three readers was 96.7 %. The average positive agreement (APA) and average negative agreement (ANA) between the three readers was 96.6 and 96.8 %, respectively (Table 5). The intra-reader (within reader) OPA was 96.3 %, the APA was 96.2 % and the ANA was 96.4 %. For HNSCC, the OPA between the three readers was 90.8 %. The APA and ANA between the three readers was 90.9 and 90.8 %, respectively. The intra-reader OPA was 94.3 %, the APA was 94.4 %, and the ANA was 94.3 %. These results met the requirement criteria for the assay.

**Table 5:** Inter- and intra-reader performance results

Study outline	Design	Inter-reader, % (95 % CI)	Intra-reader, % (95 % CI)
NSCLC cases: inter- and intra-reader agreement on a cohort previously screened for PD-L1 status	81 cases 40 PD-L1 high 41 PD-L1 low/negative	APA 96.6 % (93.8–98.8) ANA 96.8 % (93.9–98.9) OPA 96.7 % (94.2–98.9)	APA 96.2 % (92.7–98.8) ANA 96.4 % (93.0–98.8) OPA 96.3 % (93.3–98.8)
HNSCC cases: inter- and intra- reader agreement on a cohort previously screened for PD-L1 status	100 cases 50 PD-L1 high 50 PD-L1 low/negative	APA 90.9 % (86.0–94.9) ANA 90.8 % (86.0–94.8) OPA 90.8 % (86.7–94.7)	APA 94.4 % (91.1–97.1) ANA 94.3 % (91.0–97.1) OPA 94.3 % (91.3–97.0)

*ANA* average negative agreement, *APA* average positive agreement, *CI* confidence interval, *HNSCC* head and neck squamous cell carcinoma, *NSCLC* non-small cell lung cancer*, OPA* overall percentage agreement, *PD-L1* programmed cell death ligand-1

#### Inter-laboratory reproducibility

For NSCLC, the average positive and average negative inter-laboratory reproducibility was 93.3 % and 79.5 %, respectively. The OPA was 86.4 %. For HNSCC, the average positive and average negative inter-laboratory reproducibility was 89.4 and 99.5 %, respectively. The OPA was 94.5 % (Table 6). These results met the requirement criteria for the assay.

**Table 6:** Inter-laboratory performance results

Study outline	Design	Results, % (95 % CI)
Inter-laboratory reproducibility (NSCLC) (*n* = 3 labs, 2 pathologists per lab) Each case tested 5 separate times	14 cases 7 PD-L1 high 7 PD-L1 low/negative	PPA 93.3 (89.0–95.9) NPA 79.5 (73.6–84.4) OPA 86.4 (82.7–89.3)
Inter-laboratory reproducibility (HNSCC) (*n* = 3 labs, 2 pathologists per lab) Each case tested 5 separate times	14 cases 7 PD-L1 high 7 PD-L1 low/negative	PPA 89.4 (84.4–92.9) NPA 99.5 (97.3–99.9) OPA 94.5 (91.8–96.3)

*HNSCC* head and neck squamous cell carcinoma, *NPA* negative percentage agreement, *NSCLC* non-small cell lung cancer, *OPA* overall percentage agreement, *PPA* positive percentage agreement

## Discussion

IHC assays used to detect the presence of PD-L1 protein in tumor tissues are designed and needed to aid clinical treatment decisions by identifying cancer patients most likely to respond to antibody therapies that target the PD-1/PD-L1 pathway. This must be considered in the context that PD-L1 expression is complicated in a number of ways, as previously noted. Chief among these are that PD-L1 protein may localize to the cytoplasm or cell membrane, [19, 27] expression levels vary greatly among individual cells, [28, 29] and PD-L1 is expressed in tumor cells of a number of important cancer types as well as immune cells in even more cancers. In particular, PD-L1 expression is characteristic of a number of NSCLC [20, 38, 39] and HNSCC patients [18, 35, 40]. In this light, the VENTANA PD-L1 (SP263) Assay was developed to address many of these complexities and was validated to reliably detect PD-L1 protein in FFPE NSCLC and HNSCC tissue samples using a rabbit monoclonal antibody optimized for use with the OptiView DAB IHC Detection Kit.

The PD-L1 (SP263) assay showed the required level of analytical specificity and sensitivity for detecting engineered and endogenous PD-L1 protein in cell lines across a wide dynamic range of expression. Additionally, it was shown to detect PD-L1 expression levels as low as ~1,600 receptors per cell. The rabbit monoclonal antibody used in the assay was raised against the cytoplasmic region of human PD-L1 and produced a single clear band of the appropriate molecular weight in Western blot analysis. Target specificity was also demonstrated by transfecting PD-L1 into cell lines with no endogenous PD-L1 expression, producing clear staining with the PD-L1 (SP263) assay, and no staining in cells transfected with PD-L2. Antibodies raised against the cytoplasmic domain of PD-L1 have been reported to offer better visualization of membrane PD-L1 compared with those raised against the extracellular domain [22].

The assay detected PD-L1 immunoreactivity with the anticipated staining patterns in a panel of normal tissue samples with known PD-L1 expression (e.g., placental trophoblasts and immune cells) and in tumor cells from NSCLC and HNSCC tissue samples with a range of PD-L1 staining intensities and frequencies representing the heterogeneity of tissue expression. No unexpected cross-reactivity was observed with the antibody.

Numerous analytical variables have the potential to affect the reliability of assay results and the impact of these variables was extensively evaluated during assay development. In terms of preanalytical variables, the antibody demonstrated appropriate staining across all tissue section thicknesses tested and also accurately represented the PD-L1 high or PD-L1 low/negative staining status in each case. Changes in the recommended tissue fixative used (10 % NBF, zinc formalin, or Z5 [95 % alcohol, AFA, and Prefer not recommended]) over time periods ranging from 6 to 72 h and cold tissue ischemia for periods ranging from 0 to 24 h had a negligible effect on staining performance, as did cut-slide storage conditions of 2 − 8 and 30 °C for varying time periods of up to 12 months.

For assay performance over repeated measurements, the assay exceeded the 90 % pass criterion for intra-day reproducibility within a single staining run, inter-day precision (performance on the same BenchMark ULTRA instrument over 5 non-consecutive days for a minimum of 20 days) and intra-platform precision (performance across three BenchMark ULTRA instruments). The assay also exceeded the 90 % pass criteria for determination of intermediate precision across three antibody lots, three detection kit lots, and three BenchMark ULTRA platforms. Assay performance test scores (negative percentage agreement, positive percentage agreement, and OPA) were in the same range as those reported for the FDA-approved Dako PD-L1 IHC 28-8 pharmDx and PD-L1 IHC 22C3 pharmDx assays [14, 15]. Inter- and intra-reader performance scores (APA, ANA, and OPA) were all >90 % and comparable with observer-to-observer reproducibility values reported for the validated Dako PD-L1 IHC 28-8 pharmDx [52] and PD-L1 IHC 22C3 pharmDx assays [15].

A scoring algorithm for PD-L1 staining with the PD-L1 (SP263) assay was defined on the basis of an analysis of PD-L1 staining parameters observed from NSCLC and HNSCC samples from the NCT01693562 clinical trial of durvalumab, together with clinical response data. Statistical analysis of PD-L1 staining parameters in this study indicated that the parameter that correlated best with clinical response to durvalumab was PD-L1 expression in the membrane of tumor cells, regardless of staining intensity. Other PD-L1 parameters, however, remain under investigation. Samples were considered to have high expression when ≥25 % of viable tumor cells demonstrated membrane staining for PD-L1 at any intensity [1]. The scoring algorithm thus separates patients into two groups: PD-L1 high (≥25 % of tumor cells with membrane staining for PD-L1), and PD-L1 low/negative (<25 % of tumor cells with membrane staining). Separate analysis of samples from NSCLC and HNSCC cohorts independently confirmed the reliability of the ≥25 % staining cut-off and exclusion of cytoplasmic tumor cell staining, immune cell staining, and staining intensity from the analysis of PD-L1 staining status. Using this defined scoring algorithm, the classifications of patient tumor samples as PD-L1 high or PD-L1 low/negative were highly reproducible among pathologists, with an OPA of 96.7 % for NSCLC among three pathologists.

Although several different assays to determine PD-L1 positivity in tumor tissue have been developed, few have been performance tested using comparable assay conditions. There may be differences in assay performance owing to the diversity of antibody clones with different affinities, raised against different areas (epitopes) on the PD-L1 molecule (e.g., clones E1L3N, SP142, and SP263 are raised to epitopes within the intracellular domain, and clones 22C3 and 28-8 to epitopes within the extracellular domain). Furthermore, a number of associated IHC protocols have been developed, relying on differing antigen retrieval conditions and staining platforms in particular. These differences are likely to generate different staining patterns [22, 28, 55]. Tissue fixation, handling, processing, and timing of sample collection in relation to current disease and treatment stage may also influence assay outcomes. For these and other reasons, the reliability of some data reported on PD-L1 expression has been difficult to assess, and highlights the need for standardized use of well-validated PD-L1 assays for the detection and scoring of PD-L1 in patient tumor samples. A recent comparative study featuring the VENTANA PD-L1 (SP263) Assay, showed a high analytical correlation between the three different commercially available PD-L1 assays (Dako 28.8, Dako 22C3 and VENTANA SP263) [51].

The heterogeneity of PD-L1 expression also poses a challenge for pathologists [28, 29]. Unlike other biomarkers in NSCLC, PD-L1 protein expression does not give a binary signal, instead, it presents as a continuum of expression including absent, low, medium, and high levels, and also varies between tumor cells [55]; some tumors classified as PD-L1 low/negative at the biopsy site may be PD-L1 high at a different location. The creation of a binary positive or negative result for PD-L1 status is achieved using a staining cut-off. This can mean that the probability of response to anti-PD-1/PD-L1 treatment differs very little in patients with PD-L1 low/negative tumors just below the cut-off, and patients with PD-L1 high tumors just above the cut-off. The lower the staining cut-off, the greater the risk of misclassifying patients in terms of probability of response [55]. These and other concerns are currently being evaluated in ongoing clinical trials with durvalumab monotherapy and combination therapies.

This analysis demonstrated the reliability of the PD-L1 (SP263) assay to determine the PD-L1 expression of NSCLC and HNSCC tumor samples obtained in a clinical setting based on a ≥25 % cut-off value for positively stained tumor cells. Preliminary data from retrospective analyses of tumor samples obtained from a clinical trial of the anti-PD-L1 agent durvalumab, demonstrated that PD-L1 high expression, identified by the PD-L1 (SP263) assay and scoring algorithm, was a useful predictor of treatment response [1].

## Conclusions

The VENTANA PD-L1 (SP263) Assay met all of the predefined acceptance criteria (according to its proposed intended use as a clinical diagnostic test), showing analytical specificity, sensitivity, robustness and precision, and obtaining the required performance scores for day-to-day, site-to site and observer-to-observer repeatability and reproducibility, confirming its reliability for staining of FFPE NSCLC and HNSCC samples across different testing environments.

The PD-L1 (SP263) assay is currently in use in ongoing clinical trials of durvalumab, as monotherapy and in combination with tremelimumab, as part of a comprehensive clinical development program in NSCLC and HNSCC. These studies support the use of the PD-L1 (SP263) assay for testing on clinical samples and form the basis for confirming clinical utility.

## Abbreviations

AFA: Acidified formal alcohol
ANA: Average negative agreement
APA: Average positive agreement
AUC: Area under the curve
CI: Confidence interval
DAB: 3,3’-diaminobenzidine tetrahydrochloride
FACS: Flow cytometry analysis
FDA: Food and Drug Administration
FFPE: Formalin-fixed, paraffin-embedded
HNSCC: Head and neck squamo﻿us cell carcinoma
HQ: Hydroxyquinoxaline
HRP: Horse radish peroxidase
IHC: Immunohistochemistry
NA: Not applicable
NBF: Neutral-buffered formalin
NPA: Negative percentage agreement
NSCLC: non-small cell lung cancer
OPA: Overall percentage agreement
ORR: Objective response rate
PD-1: Programmed cell death-1
PD-L1: Programmed cell death ligand-1
PD-L2: Programmed cell death ligand-2
PPA: Positive percentage agreement
RECIST: Response Evaluation Criteria In Solid Tumors
ROC: Receiver operating characteristic
TM: Tumor membrane
